# Mr. Maddock, on Vaccine Inoculation

**Published:** 1801-02

**Authors:** B. Maddock

**Affiliations:** Nottingham


### *To the Editors of the Medical and Phyſical Journal.*

GENTLEMEN,

I Should be ſorry to excite prejudices in the minds of your readers, or of the public, againſt the introduction of the Vaccine Diſeaſe as a ſubſtitute for the Variolous, being well convinced that, in many reſpects, much preference is due to it, and that great credit attaches to thoſe gentlemen who have ſo meritoriouſly ſtepped forward in its ſupport; but I have to lament, that its advantages are ſomewhat over-rated, when we are told, from reſpectable authority, that it is a diſeaſe free from danger, and that, when introduced by inoculation, the ſymptoms in children are uniformly mild, from the inſertion of the matter to the period of ſcabbing. It is a great misfortune, that proſelytes to new ſyſtems do not always carefully examine into opinions handed down from high authority, but give them implicit credit; and it is equally unfortunate, that, in the recommendation of any new doctrine, the unfavourable ſymptoms are too frequently placed in the back ground, and only the more pleaſing ones expoſed to public view.

Occaſionally we hear of patients inoculated with Vaccine matter who have had the diſeaſe with ſome ſeverity, but we are not told in what the violence of it conſiſted.

In the two caſes I ſhall take the liberty of laying before the public, there are ſome circumſtances which are by no means explicable by any account I have yet heard of the diſeaſe; and though ſymptoms ſomewhat ſimilar are recorded in No. XXII. of your uſeful Medical Journal, yet, in many reſpects, the facts will be found to vary much.

In thoſe caſes the ſymptoms were extenſive eryſipelas, ſpreading rapidly from the inoculated part, accompanied by conſiderable conſtitutional affection. Theſe caſes were ſubmitted to the inſpection of Drs. Wollaſton, Pearſon, Griffiths, and Crofts, whoſe opinions were, that the unfavourable ſymptoms aroſe from the matter with which the children were inoculated having been taken at a late period of the diſeaſe, after a brown ſcab had formed, and when it had acquired a purulent appearance, and conſequently had, by degenerating, loſt the power of giving the Cow-pox infection. This opinion was alſo ſanctioned by Dr. Lettſom, who ſaw the patients.

I know many inſtances where Vaccine matter has been taken from the arms of patients as late as the eleventh and twelfth days, when it had acquired a purulent appearance, and a brown ſcab had formed, which matter has been inſerted into the arms of of children, on whom it has produced a very mild difeafe, and from whom fluid matter has been taken to inoculate others, who have alfo had the difeafe in a manner equally mild. It will be difficult to reconcile thefe facts, which are attefted not only by my own experience, but by that of fome very refpectable furgeons in this place, with the opinions advanced, that matter taken at fo late a period lofes, by degenerating, the power of producing the difeafe.

On the 22d of October, 1800, I inoculated Mr. Hulfe's child, aged fix months, with fluid matter taken on the eighth day from the arm of a child who had the difeafe very favourably. Until the eighth day, appearances were promifing, and indicated a mild difeafe; but on the ninth, the parts furrounding the incifion began to inflame, and became very hard: The inoculated part, inftead of putting on a puftular appearance, degenerated into an ulcer, and at this time much derangement of the fyftem took place, as fever, ficknefs, and great inquietude.—I applied emollient cataplafms to the arm, and dreffed the fore with equal parts of ungt. hydrarg. nitrat. and ungt. ceræ, notwithftanding the ufe of which, and warm fomentations, &c. the parts changed their appearance very little until the fixteenth day, when the inflammation extended to the fhoulder and hand, on the fingers of which, feveral vefications appeared. While thefe parts became the feat of difeafe, the inflammation completely left the inoculated part, and the ulcer acquired a healthy afpect. As the inflammation left the hand and fhoulders, it fpread to the back, breaft, and face, on the fame fide, and after remaining there about twenty-four hours, it difappeared, leaving the parts very much difcoloured, and attacked the other fide of the back and breaft, and afterwards the arm and hand; but before it became fo violent as on the fide firft affected, the child was exhaufted by want of reft and exceffive irritation, and died on the twenty-fixth day after inoculation. It is right to ftate, that on the twelfth day, the nurfe who fuckled this child, was taken ill of an inflammatory rheumatifm, and continued fo during the time it lived; and as it refufed almoft every other kind of food but the breaft milk, I fear its fymptoms might be increafed, by the neceffity we were under of allowing it the breaft to keep it quiet and give it nourifhment. Not having feen or heard of any cafe fimilar to this, I was willing to attribute the fymptoms to accidental caufes; but, a few weeks afterwards, another cafe occurred, which, from its near refemblance to this, convinced *me* they were the confequence of the Cow-pox.

On the 27th of November, I inoculated in both arms a very healthy child of Mr. Green's, of Lenton Abbey, aged five five months, with fluid taken on the ninth day from the arm of a child equally healthy. Previous to the eighth day there was no appearance which indicated any thing unfavourable. The incisions in each arm were gradually elevated into small pustules, filled with limpid matter, with a small inflammation round their base. On the ninth day the inflammation on the left arm spread very rapidly, and before the next day it occupied the whole arm, but without any vesications, as in the preceding case By the twelfth day the inflammation was much diminished on the arm, but appeared on the back, breast, and face on the same side. At this time the incision on the left arm, which, on the tenth day, had degenerated into a sordid ulcer, became almost dry, and began to form a scab, the inflammation surrounding it being intirely gone. After the inflammation had continued on these parts about twenty-four hours, it removed to the right arm, which was now dry, and had scabbed over.

It extended from the shoulder to the elbow, and also affected the right side of the body in a similar manner it had the left, and after having exercised a kind of sportive fancy on different parts of the body, for at least five weeks, a small abscess formed in the axilla, on the right side, and the child perfectly recovered. The incisions in both the childrens' arms were made very superficial and small, and the matter was used a few hours after it was taken, being first diluted with a little warm water.—Mr. Green's child was at its mother's breast, and had it not experienced the most unremitting attention, I have much reason to fear, the consequences might have proved serious. From this child's right arm I took some matter on the ninth day, with which I inoculated two children, each of whom passed through the disease as favourably as any I ever saw.

These facts are most impartially related, and no circumstance is omitted which I thought worthy attention. I believe, after all, we must refer much to idiosyncrasy; and as it frequently occurs in the variolous disease, that two children inoculated with the same matter, and apparently under similar circumstances, shall be very differently affected, so it will occasionally happen to those inoculated with Vaccine matter. I am, &c.

*Nottingham, Jan.* 8, 1801. B. MADDOCK.

